# Prevalence and incidence of pemphigus in urban Chinese: A nationwide population-based study

**DOI:** 10.1016/j.jdin.2026.04.022

**Published:** 2026-06-01

**Authors:** Liuqi Zhao, Panpan Shang, Xixue Chen, Xuejun Zhu, Hang Li, Suying Feng, Wei Li, Meng Pan, Baoqi Yang, Guiying Zhang, Chen Wei, Chaiquan Li, Siyan Zhan, Shengfeng Wang, Mingyue Wang

**Affiliations:** a Department of Dermatology, Peking University First Hospital, Beijing, China; b National Autoimmune Bullous Disease Cooperative Group, China; c National Clinical Research Center for Skin and Sexually Transmitted Diseases, Beijing, China; d Beijing Key Laboratory of Innovative Clinical Research and Translation in Skin Diseases, Beijing, China; e Institute of Dermatology, Chinese Academy of Medical Sciences, Peking Union Medical College, Nanjing, Jiangsu, China; f Department of Dermatology, West China Hospital, Rare Diseases Center, Sichuan University, Chengdu, Sichuan, China; g Department of Dermatology, Rui Jin Hospital, Shanghai Jiao Tong University School of Medicine, Shanghai, China; h Dermatology Hospital of Shandong First Medical University, Jinan, Shandong, China; i Department of Dermatology, The Second Xiangya Hospital, Central South University, Changsha, Hunan, China; j Shanghai Songsheng Business Consulting Co. Ltd, Beijing, China; k Department of Epidemiology and Biostatistics, School of Public Health, Peking University, Beijing, China; l Research Center of Clinical Epidemiology, Peking University Third Hospital, Beijing, China; m Center for Intelligent Public Health, Institute for Artificial Intelligence, Peking University, Beijing, China

**Keywords:** China, comorbidity, epidemiology, health economics, incidence, pemphigus, prevalence

*To the Editor:* Pemphigus is a rare, life-threatening autoimmune blistering disease with marked geographic variation. While data exist from Europe, North America, and parts of Asia, nationwide epidemiologic estimates for mainland China remain absent.

We conducted a retrospective, population-based study using the 2017 Chinese Urban Employee Basic Medical Insurance database, covering 122.3 million adults across 136 cities in 20 provinces (Table I, Supplementary Fig 1, available via Mendeley at https://data.mendeley.com/datasets/h3jz3jrsg7/1). Pemphigus cases were identified via international classification of diseases-10 code L10 and related diagnostic text; ambiguous records were excluded after dual dermatologist review. Incident cases required a 4-year washout period (2013-2016) without a prior pemphigus diagnosis. To estimate the national prevalence and incidence rates, province-specific rates were pooled using a random-effects meta-analysis to account for regional heterogeneity. These rates were then standardized to the 2010 Chinese census and applied to the 2017 population to estimate total patient numbers. Furthermore, economic burden and comorbidities were assessed from claims data. Detailed methods are available in Supplementary Methods, available via Mendeley at https://data.mendeley.com/datasets/h3jz3jrsg7/1.

**Table I:** Crude prevalence and incidence of pemphigus in urban China in 2017

	Population (million), *n* (%)	Pemphigus cases, *n* (%)	Crude prevalence (95% CI), per million	Incident pemphigus cases, *n* (%)	Crude incidence rate (95% CI), per million person-years
Total	122.3	3148	17.38 (10.92, 25.33)	2172	12.63 (7.89, 18.47)
Age, y (mean, SD)	42.47 (15.71)	57.38 (16.61)	-	56.90 (17.05)	-
Sex					
Male	67.3 (55.0)	1679 (53.3)	17.64 (11.67, 24.82)	1138 (52.4)	12.22 (7.95, 17.37)
Female	55.0 (45.0)	1469 (46.7)	16.70 (9.32, 26.16)	1034 (47.6)	12.84 (7.21, 20.03)
Age group					
18-29	25.9 (21.2)	145 (4.6)	2.04 (0.54, 4.22)	121 (5.6)	1.72 (0.42, 3.62)
30-39	30.2 (24.7)	392 (12.4)	6.37 (3.04, 10.75)	292 (13.4)	4.73 (2.03, 8.36)
40-49	27.0 (22.0)	563 (17.9)	12.82 (7.59, 19.31)	389 (17.9)	8.92 (5.19, 13.55)
50-59	19.1 (15.6)	645 (20.5)	21.00 (10.02, 35.74)	424 (19.5)	14.58 (7.40, 23.96)
60-69	11.9 (9.7)	613 (19.5)	39.79 (16.40, 72.77)	390 (18.0)	24.76 (9.85, 45.82)
70-79	5.5 (4.5)	425 (13.5)	67.70 (29.93, 119.72)	297 (13.7)	47.21 (21.80, 81.52)
80-89	2.4 (2.0)	327 (10.4)	115.38 (36.07, 235.60)	230 (10.60)	82.22 (25.30, 168.01)
≥90	0.3 (0.3)	38 (1.2)	42.90 (0.80, 123.83)	29 (1.3)	26.45 (0.04, 82.86)
Region					
East	52.7 (43.1)	1518 (48.2)	17.21 (5.80, 34.92)	1063 (48.9)	11.93 (3.74, 24.65)
North	13.1 (10.7)	506 (16.1)	28.01 (3.75, 74.55)	413 (19.0)	23.79 (4.27, 58.93)
North-East	11.4 (9.3)	276 (8.8)	13.00 (0.00, 55.77)	186 (8.6)	8.54 (0.00, 38.24)
North-West	5.4 (4.4)	60 (1.9)	14.83 (3.05, 34.98)	53 (2.5)	13.13 (2.30, 32.28)
South-Central	30.7 (25.1)	650 (20.6)	16.04 (4.02, 35.91)	343 (15.8)	9.46 (2.65, 20.35)
South-West	9.0 (7.4)	138 (4.4)	15.30 (12.84, 17.98)	114 (5.2)	12.64 (10.41, 15.08)

We identified 3148 prevalent and 2172 incident pemphigus cases. The standardized national prevalence of pemphigus in Chinese adults in 2017 was 22.45 (95% CI: 10.95-38.00) per million and the incidence was 15.99 (95% CI: 8.05-26.60) per million person-years, corresponding to an estimated 31,207 prevalent and 22,227 incident adults nationwide (Table I). Prevalence and incidence rates both increased progressively with age, peaking in those aged 80-89 years. No significant sex difference was observed (Supplementary Fig 2, Tables I and II, available via Mendeley at https://data.mendeley.com/datasets/h3jz3jrsg7/1). Marked regional variation was noted, with the highest rates in Northern China and the lowest in the Northeast (Supplementary Fig 3, available via Mendeley at https://data.mendeley.com/datasets/h3jz3jrsg7/1).

Pemphigus imposes a substantial economic burden, with an annual per-capita hospitalization expense of US$2086.71 (Table II). Women and younger patients incurred higher inpatient expenses, while females and middle-aged patients faced the highest outpatient costs. The mean hospital stay was 15.17 days. The most common comorbidities were asthma (2.47%), psoriasis (2.08%), and rheumatoid arthritis (1.67%) (Supplementary Fig 4, available via Mendeley at https://data.mendeley.com/datasets/h3jz3jrsg7/1).

**Table II:** Cost of pemphigus in urban China in 2017

	Total	Male	Female	18-44	45-59	≥60
Inpatient cost						
Patient number, *n* (%)^a^	556	328 (59.0)	228 (41.0)	72 (13.0)	162 (29.0)	322 (58.0)
Annual per-capita cost, $	2086.71	1969.87	2255.18	2255.70	1980.83	2101.96
Per-episode cost, $	1587.52	1536.88	1656.27	1779.50	1306.85	1717.33
Annual per-capita hospitalization, *n*	1.31	1.28	1.36	1.27	1.52	1.22
Length of stay, d	15.17	14.44	16.12	15.38	13.56	16.11
Outpatient cost						
Patient number, *n* (%)^b^	2490	1283 (51.5)	1207 (48.5)	686 (27.6)	782 (31.4)	1022 (41.0)
Annual per-capita cost, $	213.76	206.83	221.13	164.64	243.84	223.68
Per-episode cost, $	61.31	60.48	62.16	55.27	61.95	64.22
Annual per-capita visit, *n*	3.49	3.42	3.56	2.98	3.94	3.48

Medical costs were extracted from UEBMI claims data and converted to USD using the 2017 average CNY-to-USD exchange rate. Patients with missing or conflicting cost records were excluded for cost calculation, accounting for (a) 8.4% of the inpatient and (b) 2.0% of the outpatient samples, respectively.

*UEBMI*, Urban Employee Basic Medical Insurance.

This first nationwide study establishes pemphigus as a rare disease in urban Chinese mainland. The prevalence is comparable to that in Taiwan region^1^ but lower than in Western countries.^2^^,^^3^ The annual inpatient cost (US$2087) exceeds the national average (US$1629),^4^ and the mean hospital stay (15.2 days) is twice that of the United States (7.2 days),^5^ highlighting a substantial economic burden. Several limitations exist. First, case identification relied on administrative international classification of diseases-10 codes without immunopathologic confirmation, and this coding strategy lacks formal validation for pemphigus in the Urban Employee Basic Medical Insurance database. Second, the inability of these codes to specify pemphigus subtypes limits diagnostic precision. Finally, extrapolating national estimates from the Urban Employee Basic Medical Insurance database assumes uniform disease prevalence across socioeconomic strata. As this database only includes urban employees, it may not reflect the true burden among rural or unemployed adults, limiting the generalizability of our findings. Despite these caveats, this nationwide study establishes pemphigus as a rare disease with profound economic burden in China, providing essential baseline data to guide clinical decision-making and health resource allocation.

## Conflicts of interest

None disclosed.
